# Lower trunk motion and speed-dependence during walking

**DOI:** 10.1186/1743-0003-6-9

**Published:** 2009-04-09

**Authors:** Justin J Kavanagh

**Affiliations:** 1 School of Physiotherapy and Exercise Science, Griffith Health, Griffith University, Gold Coast, Queensland, Australia

## Abstract

**Background:**

There is a limited understanding about how gait speed influences the control of upper body motion during walking. Therefore, the primary purpose of this study was to examine how gait speed influences healthy individual's lower trunk motion during overground walking. The secondary purpose was to assess if Principal Component Analysis (PCA) can be used to gain further insight into postural responses that occur at different walking speeds.

**Methods:**

Thirteen healthy subjects (23 ± 3 years) performed 5 straight-line walking trials at self selected slow, preferred, and fast walking speeds. Accelerations of the lower trunk were measured in the anterior-posterior (AP), vertical (VT), and mediolateral (ML) directions using a triaxial accelerometer. Stride-to-stride acceleration amplitude, regularity and repeatability were examined with RMS acceleration, Approximate Entropy and Coefficient of Multiple determination respectively. Coupling between acceleration directions were calculated using Cross Approximate Entropy. PCA was used to reveal the dimensionality of trunk accelerations during walking at slow and preferred speeds, and preferred and fast speeds.

**Results:**

RMS acceleration amplitude increased with gait speed in all directions. ML and VT trunk accelerations had less signal regularity and repeatability during the slow compared to preferred speed. However, stride-to-stride acceleration regularity and repeatability did not differ between the preferred and fast walking speed conditions, partly due to an increase in coupling between frontal plane accelerations. The percentage of variance accounted for by each trunk acceleration Principal Component (PC) did not differ between grouped slow and preferred, and preferred and fast walking speed acceleration data.

**Conclusion:**

The main finding of this study was that walking at speeds slower than preferred primarily alters lower trunk accelerations in the frontal plane. Despite greater amplitudes of trunk acceleration at fast speeds, the lack of regularity and repeatability differences between preferred and fast speeds suggest that features of trunk motion are preserved between the same conditions. While PCA indicated that features of trunk motion are preserved between slow and preferred, and preferred and fast speeds, the discriminatory ability of PCA to detect speed-dependent differences in walking patterns is limited compared to measures of signal regularity, repeatability, and coupling.

## Background

As the upper body accounts for a large proportion of total body mass and the bipedal base of support is continually changing, the central nervous system (CNS) is challenged to adjust motor output to remain upright and stable during walking. Perhaps the greatest challenge to regulating upper body motion is the potentially perturbing events that surround the foot contacting the ground. In particular, following foot contact the upper body tends to rotate forward and over the stance leg causing rapid stride-to-stride horizontal accelerations of the trunk [1]. In the plane of progression, erector spinae activity [2,3] and almost equal and opposite hip extensor moments [4] are generated to prevent unbalancing of the trunk early in the stance phase. In contrast, the primary source of lateral balance is proposed to be foot placement and medial-lateral moments generated about the ankle, with secondary contributions from the hip abductors to ensure trunk orientation [5]. These balance mechanisms combined with coordinated motion of torso and pelvis rotation, and arm and leg swing, cause the upper body to oscillate in a rhythmical and semi-predictable manner during unperturbed walking [6,7]. The general ability to regulate balance during walking is reflected in the acceleration profile of upper body motion [1]. In particular, acceleration patterns of a segment in close proximity to the body's centre of mass (i.e. the lower trunk) have provided valuable insight about how balance is maintained in health and disease [8].

It is generally recognised that altering the speed that an individual walks is reflected by systematic changes in temporal, kinematic, and kinetic parameters measured for the lower limb. However, considerably less is known about how gait speed influences upper body motion during walking. Of the limited data available, two contrasting observations concerning the speed-dependent responses of trunk accelerations have been identified. Firstly, increasing gait speed corresponds to increases in movement amplitude across the gait cycle. For instance, at a range of self-imposed slow to fast speeds, walking speed corresponds to an almost linear increase in the RMS amplitude of lower trunk accelerations [9,10]. Alternatively a U-shaped response in movement amplitude may be observed, where suboptimal responses are evident at an individual's non-preferred walking speeds. Using the ratio of even to odd signal harmonics, the rhythmicity of 3D lower trunk accelerations for healthy individuals have been reported to be greatest at the self-selected preferred walking speed and step frequency, with declines in rhythmicity occurring at non-preferred speeds [11]. The abovementioned discrepancy in observations using RMS acceleration and harmonic ratio highlights the need to employ a battery of tests to reveal speed-dependent postural processes.

A dichotomy many investigators face is determining whether inter- and intra-individual differences in motor output are an inherent property of the neuromuscular system, or simply due to variations in gait speed. As such, insights into postural responses are being gained with analyses that have less emphasis on the amplitude of segmental and joint motion, and more emphasis on the spatial and temporal variability of movement in relation to walking speed [12,13]. In an effort to understand how the CNS regulates motor output for a given task, importance should not only be placed on the degree of variability, but also the structure of movement variability [14]. An increasing number of motor control studies are exploring the structure of movement variability, discovering that hidden features in motor output previously believed to be noise are actually meaningful data that relates to functionality of the system. One such analysis that reveals underlying structure within a data set is Principal Component Analysis (PCA). PCA has typically been used as a dimensionality reduction tool, with a view of decreasing redundant information in multidimensional data sets by representing the original data as a few orthogonal Principal Components (PC's) [15,16]. However if PCA is applied to univariate data sets such as trunk accelerations, the extracted PC's will reveal patterns that are embedded in the waveforms of the original data set. If a relatively small number of PC's explain the majority of variance in a data set grouped across different walking speeds, then common components of walking variability exist across walking speeds.

The primary purpose of this study was to examine how gait speed influences healthy individual's trunk motion during overground walking. Lower trunk accelerations in the anterior-posterior (AP), mediolateral (ML) and vertical (VT) direction were examined using analyses that address the amplitude of motion as well as the stride-to-stride structure of trunk accelerations. It was anticipated that the amplitude of accelerations would increase systematically with walking speed. However, the structure of acceleration profiles in terms of rhythmicity, repeatability and regularity would be greatest at an individual's self-selected preferred walking speed. The secondary purpose was to perform a preliminary study to assess if PCA can be used to gain further insight into postural responses that occur at different speeds during overground walking. It was expected that a relatively small number of PC's would explain the majority of variance in lower trunk acceleration across different walking speeds.

## Methods

### Subjects

Thirteen healthy subjects (7 male, 6 female, age: 23 ± 3 years, height: 1.71 ± 0.11 m, mass: 71 ± 11 kg) with no history of musculoskeletal pathology or injury were recruited from the university community. Written informed consent was obtained from each subject prior to testing. All experimental procedures complied with the guidelines of the Griffith University Ethics Committee for Human Research.

### Experimental protocol

Subjects were required to perform 5 straight-line walking trials along a 30 m level walkway at self-selected slow, preferred, and fast walking speeds. The sequence in which walking speeds were performed was counter-balanced by randomly allocating each subject with a walking sequence; e.g. fast followed by slow followed by preferred speed walking trials. For the preferred speed, subjects were instructed in colloquial language to 'walk at a normal comfortable speed that you would use in everyday life'. For the slow and fast speeds, subjects were instructed to 'walk at a pace much slower/faster than you normally walk at, but not so slow/fast that you feel unsteady and may lose balance'.

Although head orientation and gaze fixation were not the focus of this study, subjects were encouraged to avoid looking around the laboratory, as excessive head motion will affect natural thoracic and pelvic movement patterns. Furthermore, in an effort to represent the natural oscillatory properties of the trunk, subjects performed the walking task unshod to avoid artificially damping oscillations that arise from foot contact events. Gait velocity was monitored using 3 pairs of Omron (E3JK-R4M2) photoelectric light gates spaced at 5 m intervals along the middle of the walkway. Walking trials were accepted for inclusion if gait velocity was with ± 5% for each 5 m interval recorded using the light gate system. Trials not meeting this criterion were re-performed.

### Instrumentation

Three triaxial accelerometers (Crossbow CXL02LF3, range ± 2 g) were used to measure AP, VT, and ML accelerations of the lower trunk and shanks during walking. A single accelerometer was fixed with rigid sports tape over the L3 spinous process, a region suggested to have low transverse plane rotation relative to axial rotation of the pelvis and thorax [17]. The other two accelerometers were attached to the left and right legs with sports tape, 3 cm proximal to the lateral malleolus. Before testing, subjects were encouraged to walk around the laboratory at a non-specific speed and duration until they confirmed that they felt comfortable wearing the apparatus. Prior to all testing sessions, each accelerometer axis was statically calibrated using a horizontal reference surface to ensure vertical axis output was -1 g and horizontal axes outputs were 0 g. Accelerometer data were sampled at 512 Hz using a portable data logger (Valitec AD2012 Ready DAQ) which was attached to a waist belt worn by the subject.

### Data analysis

Analog data were downloaded from the data logger onto a PC using Valitec configuration and analysis software (Version 2.5) and analysed using custom Matlab software (MathWorks, Version 6.0). Data were low-pass filtered using dual-pass zero-lag Butterworth filter with a cut-off frequency set at 20 Hz.

Following data collection, a tilt correction was applied to all acceleration data to account for any deviation in accelerometers axes from global vertical and horizontal planes whilst attached to the subject's body. The degree of axes misalignment was determined from acceleration data collected during quiet stance prior to each walking trial as per Kavanagh et al. [6]. Under static conditions, the output of each accelerometer reflects the degree of tilt in the device, which can be determined, and corrected for, using basic trigonometry. After tilt correction, axes from all accelerometers corresponded to the global AP, VT and ML axes with the subject standing in anatomical position.

### Foot contact detection

Accelerometers were fixed to the lower shank for the purpose of identifying foot contact. The site of attachment has minimal underlying subcutaneous tissue, and is close to the point of impact. Reducing the distance between the point of impact and the attachment site on the body will enhance accuracy of foot contact detection, as there is minimal opportunity for the musculoskeletal system to attenuate the acceleration signal.

**Figure 1:**
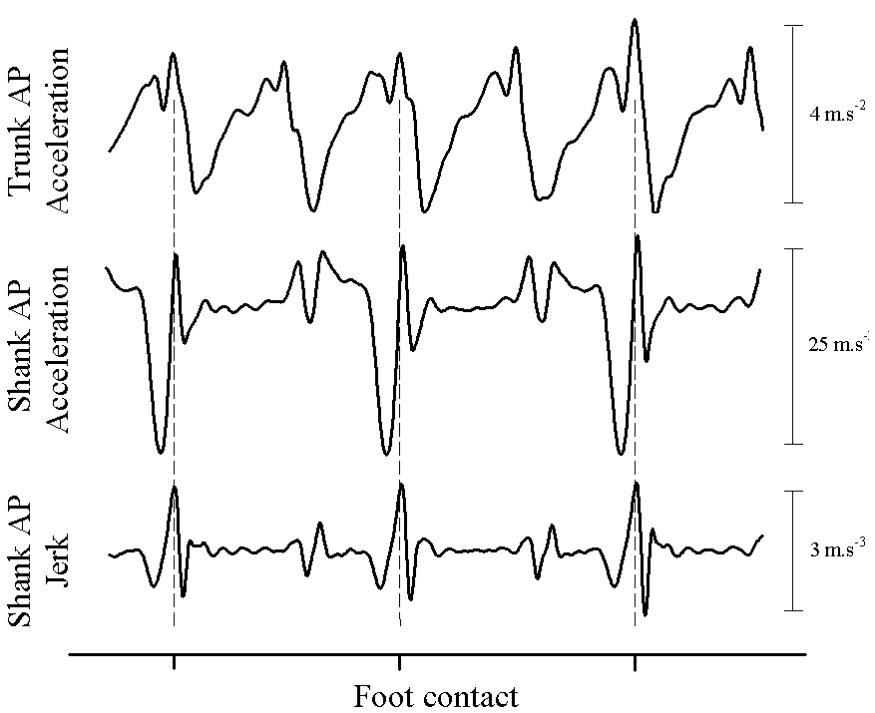
**Representative data illustrating the temporal relationship between raw trunk and shank acceleration, and shank jerk in the anterior-posterior (AP) direction**. Dashed vertical lines indicate foot contact calculated from peaks in shank jerk profile.

Foot contact was calculated from the acceleration zero crossing following peak negative accelerations for the shank in the AP direction (Figure 1). Raw AP accelerations of the shank were differentiated to produce jerk, from which an algorithm was applied to identify peaks in the signal (Figure 1). A similar foot contact peak was observed for the jerk profile for the ML acceleration of the shank, however it was lower in amplitude and more difficult to extract compared to the AP acceleration. It should be noted that peaks in the AP jerk profile correspond to peak trunk accelerations in the AP direction, which have previously been used to determine foot contact events during walking [18,19]. Data were divided into step cycles, defined as the period between left and right foot contact events (and vice-versa for the subsequent step). Two consecutive steps constituted a stride. The middle 20 step cycles were the basis of data analysis in the current study.

### Root mean square acceleration

The amplitude of trunk accelerations during each trial were assessed using RMS amplitude computed over individual strides.

### Signal repeatability

Acceleration repeatability was assessed in the present study by calculating the adjusted Coefficient of Multiple Determination (CMD), which indirectly quantifies the percentage variance accounted for within the data [20,21]. While the CMD has previously been used to report reliability of gait data collected from different testing sessions, the present study calculated CMD between strides for individual trials as a measure of stride-to-stride repeatability [22]. Waveforms that are similar return CMD values that approach 1, whereas dissimilar waveforms result in the CMD values approaching zero [21]. As the CMD is influenced by the magnitude of the signals under examination, and acceleration amplitude is correlated to walking speed, raw trunk acceleration data were normalised to each subjects preferred speed RMS acceleration.

### Signal regularity

The degree of acceleration signal regularity within each stride was determined using Approximate Entropy (ApEn). ApEn is a probability statistic based on the logarithmic likelihood that a sample of data will remain within a tolerance window defined as 20% of the standard deviation (r = 0.2) in subsequent data increments of one data point (m = 1) within a serial signal [23,24]. ApEn analysis returns a scalar value which approaches zero with increased signal regularity, and approaches two with increased signal irregularity [23,25]. Increased regularity in a signal, or a signal containing a large degree of repeatable pattern features such as a pure low frequency sine wave, will return a low ApEn value. In contrast, an irregular signal where time series events are unrelated to previous event (such as white noise) ApEn will return a high value. Similar to the CMD, as ApEn is influenced by the magnitude of the accelerations, the amplitude of raw trunk acceleration data were normalised to each subjects's preferred speed RMS acceleration.

### Directional coupling

The degree of coupling between movement directions within each stride was calculated by applying Cross ApEn to trunk acceleration signals in each direction (ie. VT-AP, VT-ML, and AP-ML coupling) [26,27]. Cross ApEn quantifies the degree of signal regularity between standardised serial signals. While Cross ApEn has similarities with correlation analysis, there is evidence to suggest that the former is more sensitive to identifying and grading subtle serial signal evolutions [24,28]. A higher Cross ApEn value is indicative of weaker coupling between paired acceleration signals, whilst a value that approaches zero indicates a stronger degree of signal congruence and coupling [24,28].

### Principal component analysis

The most common applications of PCA have been with large multivariate data sets, where the number of variables that are required to explain a biological process are reduced whilst retaining much of the variation present in the data set [16]. The reduced set of variables (PC's) are orthogonal and uncorrelated, indicating that each PC represents a different dimension present in the data. In the present study, PCA was used with a view of identifying the main sources of step-to-step variance for gait-related trunk accelerations. The procedures used to perform PCA on continuous serial data collected during human walking have been outlined in detail [15,29-31], and will only be briefly described here.

Raw acceleration data were divided by steps and normalised to 151 points. Two 150 × 151 column vector matrices were created for each subject and acceleration direction that represented trunk accelerations grouped for the slow and preferred walking speeds, and the preferred and fast walking speeds. In this study, 20 steps from each of the subjects 5 trials were used as the basis of the PCA calculations. Trunk accelerations in the AP and VT directions are biphasic during the stride cycle and similar in profile when raw data is divided into steps (for example of AP direction refer to Figure 1). In contrast, accelerations in the ML direction are monophasic during the stride cycle [6,7], and dividing accelerations into steps results in half of the raw data being positive (eg left step) and the remaining data being negative (eg right step). Therefore, accelerations that were negative during the step cycle in the ML direction were inverted so that all ML accelerations in the PCA were consistent in profile.

Following the construction of the acceleration data matrix, a mean-adjusted covariance matrix was calculated, which was the basis of the PCA. In the present study, the covariance matrix was favoured to the correlation matrix; the latter of which is more appropriate when the variables under consideration have different scaling or units [32,33]. To extract PC's, eigenvector decomposition was performed on the covariance matrix. PC's were ordered so that the variance (eigenvalues) exhibited in PC_1 _> variance PC_2 _> variance PC_3 _> ...variance PCn, where n is equal to the number of variables in the original association matrix. Often, extracted PC's contain multiple peaks and variance components which make interpretation of results difficult, or even impossible [16]. Therefore, an orthogonal Varimax rotation procedure was applied to the original PCA solution. Varimax rotation has the effect of amplifying higher loadings and suppressing lower loadings so that PC's have a simpler structure [16], often resulting in a reduced number of peaks in the extracted eigenvectors. Although a large number of PC's were extracted, only a few were retained for subsequent analysis. In the present study, a PC was only retained if its eigenvalue accounted for greater than 1% of variance in the acceleration data set.

### Statistical analysis

All statistical analyses were performed in SAS for Windows (Release 9.1). Using the Mixed procedure and Contrast statement, two-way Analysis of Variance (ANOVA) was used to determine whether the dependent variables of RMS acceleration, CMD, ApEn, and Cross ApEn were different according the speed of walking (slow, preferred, fast). Speed by direction (VT, AP, ML) interactions were selectively examined according to direction using planned contrasts applied to incremented walking speed conditions. For example, contrasts were used to identify if accelerations in the VT direction were different between slow and preferred speeds, and preferred and fast speeds for each dependent variable. Main effects were not reported in the present study, as averaging dependent variables into a walking speed condition, or alternatively the acceleration direction, reduces the capacity to interpret how the dependent variables were influenced by different walking speeds. Using the GLM procedure, MANOVA were used to determine if PC's differed between grouped walking speed conditions (slow-preferred, preferred-fast). As individual PC's are uncorrelated, each PC was entered into the MANOVA as a separate dependent variable. In the event of a significant main effect of walking speed condition or acceleration direction, post hoc analyses were performed. The level of significance for all statistical analysis in the present study was 0.05.

## Results

### Basic gait parameters

Data for gait velocity, stride duration, cadence and step length for each walking speed condition are presented in table 1. A significant main effect for speed was identified for all basic gait parameters. The average gait velocity, cadence, and step length increased from the slow to preferred to fast walking speed conditions, whereas stride duration decreased across the same conditions.

**Table 1:** Basic gait parameters for self-selected slow, preferred and fast walking speeds (mean ± SD).

Variable	Walking condition			Effect of speed
	Slow	Preferred	Fast	
Gait velocity (m.s^-1^)	0.93 ± 0.11	1.32 ± 0.18	1.78 ± 0.29	*F*(2, 24) = 443.32, *p *< 0.01)
Stride duration (s)	0.63 ± 0.05	0.53 ± 0.03	0.47 ± 0.03	*F*(2, 24) = 444.62, *p *< 0.01)
Cadence (steps.min^-1^)	95.72 ± 8.59	111.85 ± 6.69	126.49 ± 7.53	*F*(2, 24) = 519.11, *p *< 0.01)
Step length (m)	0.59 ± 0.06	0.71 ± 0.09	0.84 ± 0.11	*F*(2, 24) = 352.35, *p *< 0.01)

### Root mean square acceleration

**Figure 2:**
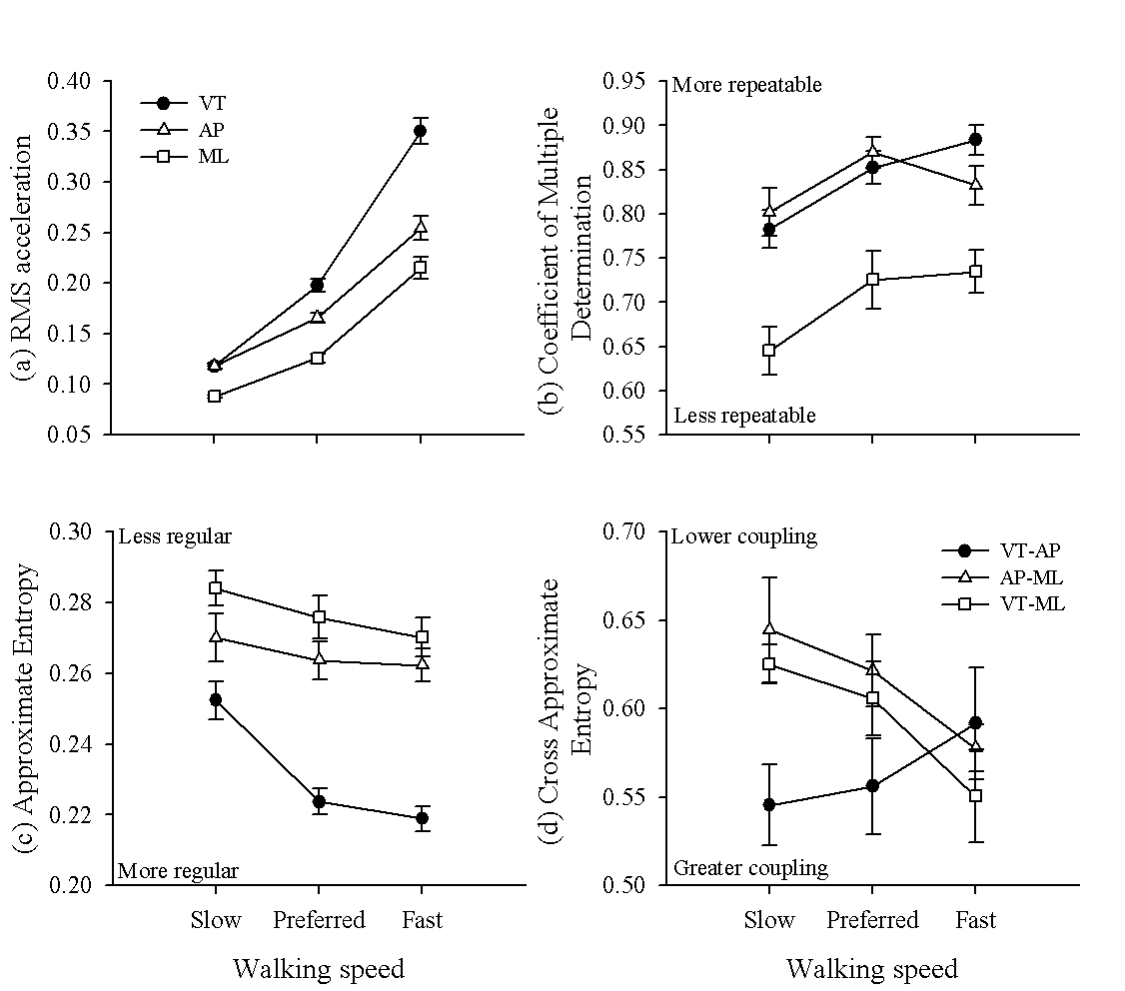
**RMS acceleration (a), Coefficient of Multiple Determination (b), and Approximate Entropy (c), derived from lower trunk accelerations in the vertical (VT), anterior-posterior (AP), and mediolateral (ML) direction**. Cross Approximate Entropy (d) representing strength of coupling between accelerations in the vertical and anterior-posterior (VT-AP), anterior-posterior and mediolateral (AP-ML), and vertical and mediolateral (VT-ML) directions. All data is presented for slow, preferred, and fast walking speeds. Error bars represent one standard error of the mean.

A significant interaction effect of walking speed and acceleration direction was identified for RMS acceleration (*F*(8, 96) = 189.92, *p *< 0.01). Contrasts revealed that RMS acceleration was greater for the preferred speed compared to the slow speed for the VT (*F*(1, 96) = 87.09, *p *< 0.01), AP (*F*(1, 96) = 30.47, *p *< 0.01), and ML directions (*F*(1, 96) = 19.56, *p *< 0.01), and greater for the fast speed compared to the preferred speed for the VT (*F*(1, 96) = 319.59, *p *< 0.01), AP (*F*(1, 96) = 108.20, *p *< 0.01), and ML direction (*F*(1, 96) = 110.54, *p *< 0.01, Figure 2a).

### Signal repeatability

A significant interaction effect of walking speed and acceleration direction was identified for CMD (*F*(8, 96) = 189.92, *p *< 0.01). Contrasts revealed that CMD was greater for the preferred speed compared to the slow speed for the VT (*F*(1, 96) = 6.00, *p *= 0.01) and ML directions (*F*(1, 96) = 7.99, *p *< 0.01, Figure 2b).

### Signal regularity

A significant interaction effect of walking speed and acceleration direction was identified for ApEn (*F*(8, 96) = 39.03, *p *< 0.01). Contrasts revealed that ApEn was lower for the preferred speed compared to the slow speed for the VT (*F*(1, 96) = 3.99, *p *= 0.04), AP (*F*(1, 96) = 5.95, *p *= 0.02) and ML directions (*F*(1, 96) = 21.24, *p *< 0.01, Figure 2c)

### Directional coupling

A significant interaction effect of walking speed and acceleration direction was identified for Cross ApEn (*F*(8, 96) = 3.21, *p *< 0.01). Contrasts revealed that Cross ApEn was lower for the fast walking speed compared to the preferred walking speed for VT-ML coupling (*F*(1, 96) = 4.82, *p *= 0.04, Figure 2d).

### Principal component analysis

**Figure 3:**
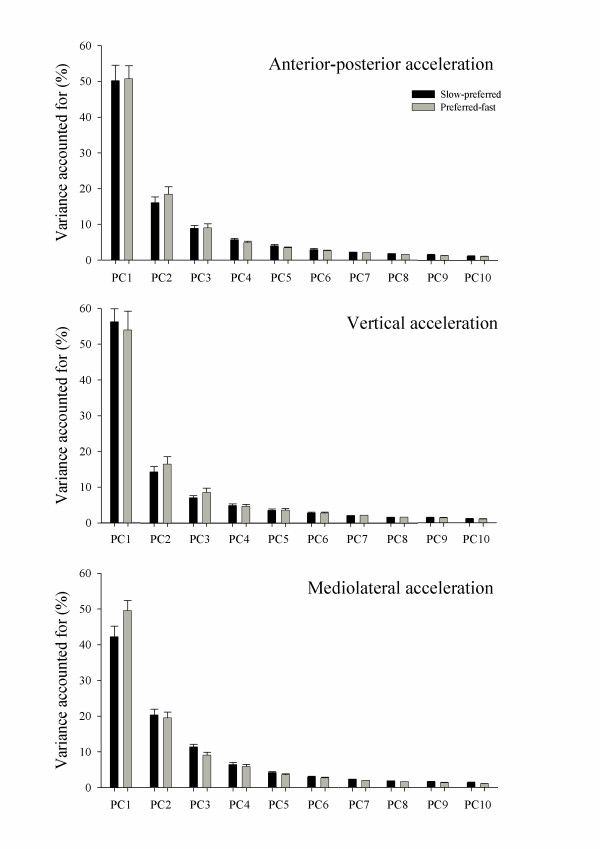
**Percentage of variance accounted for by the first ten Principal Components (PC) of lower trunk accelerations measured for the vertical, anterior-posterior, and mediolateral directions**. Error bars represent one standard error of the mean.

The number of PC's retained for analysis were 10, 10, and 11 for both the slow-preferred speed data and the preferred-fast speed data in the VT, AP and ML directions respectively. For the slow-preferred speeds the retained PC's accounted for 95.2 ± 1.1%, 94.8 ± 1.1% and 95.6 ± 1.0%, and for the preferred-fast speed the retained PC's accounted for 96.3 ± 1.0%, 95.2 ± 1.2% and 97.2 ± 1.0% in the VT, AP and ML directions respectively. No significant differences were identified for PC data between grouped walking speed conditions. Normalised eigenvalues calculated for each PC for the VT, AP and ML directions are presented in Figure 3.

**Figure 4:**
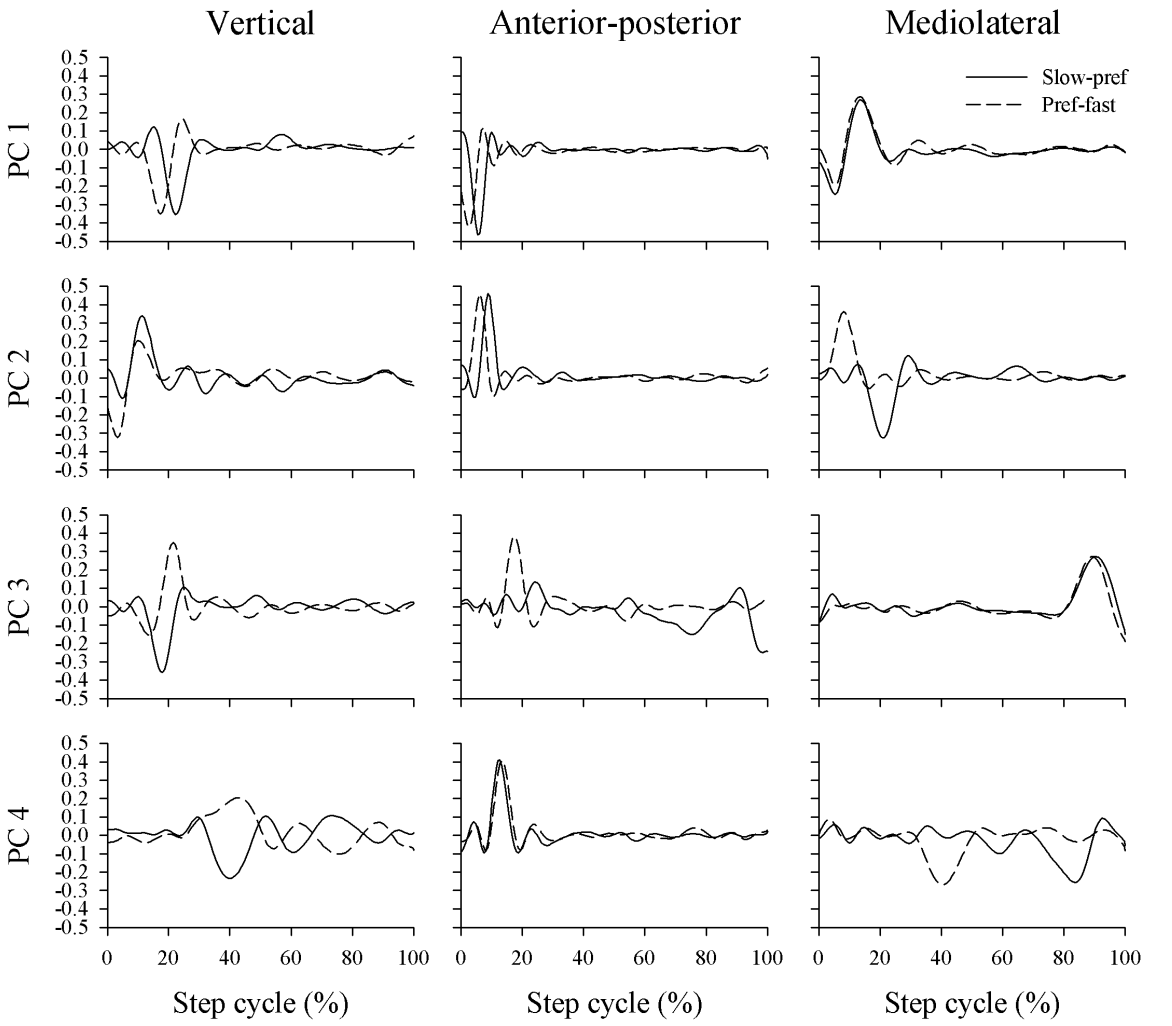
**Representative Principal Components (PC) for a single subject plotted across the step cycle**. Data represents PC's extracted from lower trunk accelerations measured for the vertical, anterior-posterior, and mediolateral directions. Solid lines are PC's extracted from grouped data for slow and preferred walking speeds, and dashed lines are PC's from grouped preferred and fast walking speeds.

Representative eigenvectors for a single subject are presented in Figure 4, illustrating the profiles of PC1 to PC4 for the slow-preferred walking speed data and the preferred-fast walking speed data. In general, peak PC's predominantly occurred between 0–20% and 80–100% of the step cycle for each direction. Peaks emerged between 20–80% of the step cycle only at higher PC's (e.g. > 6) where the variance accounted for in the acceleration data was relatively low compared to the first few PC's.

## Discussion

The primary purpose of this study was to examine how gait speed influences healthy individual's trunk motion during overground walking. Lower trunk accelerations were examined using analyses that address the amplitude of motion as well as the stride-to-stride structure of trunk accelerations. This investigation was also a preliminary study to assess if PCA can be used to gain further insight into postural responses that occur at different speeds during overground walking.

The basic gait parameters of gait velocity, cadence, and step length systematically increased from the slow to preferred to fast walking speed conditions, and as could be expected stride duration decreased during the same conditions. The different walking speeds were also found to alter the absolute amplitude of trunk, as RMS acceleration increased with gait velocity in the AP, VT, and ML directions. RMS amplitude of trunk accelerations may be a reasonable indicator of the inertial properties of the upper body that must be overcome during walking, however it provides limited insight to how motor output is regulated [11].

A noteworthy finding in the present study was that trunk accelerations in the ML and VT directions had less signal regularity and repeatability during the slow walking speed compared to preferred walking speed. However, stride-to-stride acceleration signal regularity and repeatability did not statistically differ between the preferred and fast walking speed conditions. These findings are in contrast to the U-shaped response in movement amplitude that was expected with changes in walking speed. As the fundamental requirements of human walking include progression, support and balance, marked reductions in the speed of progression may have produced added challenge to the individuals support and balance mechanisms [34]. Under normal walking conditions, mechanical models and biological data indicate that lateral balance is an active rather than passive control process, which results in greater motor output variability than the AP direction [35-37]. Therefore, the less repeatable and regular accelerations at slow speeds may be a factor of altered motor activity and variability in sensorimotor mechanisms that facilitate frontal plane balance.

In light of the greater amplitude of trunk acceleration for the fast speed compared to the preferred speed, the lack of regularity and repeatability differences between the same conditions suggest that features of trunk motion are preserved at speeds that are faster than preferred. However this finding should be taken with caution, as the subjects in the present study were walking at self-selected fast speeds. Increasing speed to beyond what is perceived as a comfortable level by the subjects would most likely result in added perturbation to the upper body. The results of the Cross ApEn analysis between acceleration directions revealed a change in coordination dynamics that assisted in controlling trunk motion at fast speeds. At the fast walking speed the coupling between accelerations in the ML and VT direction increased, thus placing a greater importance on regulating the global motion of the trunk in the frontal plane rather than independently regulating accelerations according to direction. Similar coupling dynamics have been reported for gait-related head accelerations in healthy older individuals, presumably to assist in maintaining head stability in the presence of reduced postural control [26].

Acceleration RMS amplitude, regularity, repeatability, and coupling were used collectively to characterise the speed-dependent trunk motion across the gait cycle. However, additional information about how motor output is regulated can be gained by examining the complexity of motor output [38]. In regards to gait-related trunk motion, there is evidence to suggest that the variability and complexity of trunk acceleration signals corresponds to an individual's health status. Post-stroke hemiplegic patients [39], Parkinsonian patients [40], and healthy older individuals [41] have greater complexity in gait-related trunk motion than younger individuals, which suggests that increased dimensionality may be a characteristic of reduced postural stability.

In the current study, a criterion was set where PC's were only considered for analysis when their variance was greater than 1% of the total variance in the lower trunk accelerations. Consequently, 10 PC's for the AP, 10 PC's for the VT, and 11 PC's for the ML direction were retained in the analysis. The number of PC's required to account for greater than 95% of variance was relatively low compared to the 300 dimensional set of absolute possibilities. The interpretation of dimensionality within a univariate data set is one of conjecture, partly because measurements involving a small number of biomechanical degrees of freedom (such as a single accelerometer) are reflective of dimensional organisation at other levels of the control system [42]. Sanger [43] examined the trajectory of hand motion when performing a practiced tracking task in a Cartesian coordinate system, and found that the smooth planar movement associated with copying a target trajectory was low-dimensional. It was suggested that the low-dimensional output may be a strategy to simplify the interaction between CNS control and musculoskeletal mechanics. However, the author stressed that such conclusions were speculative as applying PCA to the output of a motor task only provides a description of movement and not causality of movement. The absence of significant differences between the slow-preferred and preferred-fast PC data in the present study suggests that variance within the step cycle was similar at different walking speeds, and features of trunk motion are retained despite changes in walking speed. However, the discriminatory ability of PCA to detect speed-dependent differences in walking patterns appears to be limited compared to measures of signal regularity, repeatability, and coupling. Interestingly, PC peaks generally occurred following foot contact, suggesting that variance is greatest during the weight acceptance phase of walking. To date, upper body motion is yet to be examined in detail regarding how acceleration of the trunk is influenced by foot contact events or the swing and stance phase.

## Conclusion

The main finding of this study was that walking at speeds slower than preferred primarily alters lower trunk accelerations in the frontal plane. Despite greater amplitudes of trunk acceleration at fast speeds, the lack of regularity and repeatability differences between preferred and fast speeds suggest that features of trunk motion are preserved between the same conditions. This was partly due to an increase in coupling between accelerations in the ML and VT direction, which places a greater importance on regulating the global motion of the trunk in the frontal plane instead of independently regulating accelerations according to direction. PCA provided useful insight to the dimensionality of lower trunk motion, identifying that a relatively low number of PC's explained the majority of variance in acceleration data across different walking speeds. Further research needs to be undertaken to determine if a higher number of PC's correlates to conditions of postural instability. If this is the case, PCA may be a tool that can be employed in a rehabilitation setting to monitor improvement or declines in postural control.

## Competing interests

The author declares that he has no competing interests.
